# Review on Interfacial Bonding Mechanism of Functional Polymer Coating on Glass in Atomistic Modeling Perspective

**DOI:** 10.3390/polym13142244

**Published:** 2021-07-08

**Authors:** Hyunhang Park, Sung Hoon Lee

**Affiliations:** Corning Technology Center Korea, Corning Precision Materials Co., Ltd., 212 Tangjeong-ro, Asan 31454, Chungcheongnam-do, Korea; hyunhangpark@corning.com

**Keywords:** polymer film, adhesion, bonding mechanism, surface morphology, relative humidity

## Abstract

Atomistic modeling methods are successfully applied to understand interfacial interaction in nanoscale size and analyze adhesion mechanism in the organic–inorganic interface. In this paper, we review recent representative atomistic simulation works, focusing on the interfacial bonding, adhesion strength, and failure behavior between polymer film and silicate glass. The simulation works are described under two categories, namely non-bonded and bonded interaction. In the works for non-bonded interaction, three main interactions, namely van der Waals interaction, polar interaction, and hydrogen bonds, are investigated, and the contributions to interfacial adhesion energy are analyzed. It is revealed that the most dominant interaction for adhesion is hydrogen bonding, but flexibility of the polymer film and modes of adhesion measurement test do affect adhesion and failure behavior. In the case of bonded interactions, the mechanism of covalent silane bond formation through condensation and hydrolysis process is reviewed, and surface reactivity, molecular density, and adhesion properties are calculated with an example of silane functionalized polymer. Besides interfacial interactions, effects of external conditions, such as surface morphology of the glass substrate and relative humidity on the adhesion and failure behavior, are presented, and modeling techniques developed for building interfacial system and calculating adhesion strengths are briefly introduced.

## 1. Introduction

Thin polymer films play critical roles in various glass industry applications. They have been used as adhesives in automotive and architecture, an anti-fouling coating layer in touch-screen applications, a substrate for organic light emitting diode, and a protective layer for glass packaging [1,2,3,4,5,6]. Required interfacial properties are widely varied depending on the purpose of the coating on the surface. For example, anti-fouling layer requires the hydrophobic/oleophobic property at the interface with high adhesion strength for durability on glass surface [7]. On the other hand, polyimide thin film as a carrier for display glass needs a moderate level of adhesion with the glass surface, which can prevent failure at the interface, as well as allow easy detachment if needed [8,9]. In the case of a glass-surface protection layer, adhesion of polymer layer on the glass is sufficient so long as it can prevent stiction of dust particles or stains. However, the layer should be completely removable by a simple process, such as washing, for easy handling after transportation. Consequently, understanding the interfacial behavior is of primary importance for the development of polymer coating for target goals.

Generally, polymer films are easily deformed, and thereby can form many kinds of interfaces with physical and chemical interactions [10,11,12,13]. For instance, polymeric chains can be diffused into the porous and irregular surface of the substrate and entangle with each other, leading to a mechanically interlocked interface. In cases of epoxy resin or rubber materials, additional heating may induce crosslinking between entangled chains to form harder locking. If surface species of the counterpart substrate have high mobility, an interdiffusion layer can also be formed. However, typical interfacial interactions explained above are significantly limited by surface properties of the glass substrate. It is because polymer chains are hard to diffuse into the glass surface without additional engineering due to low surface roughness and porosity, as well as high cohesive strength of glass [14]. In addition, the strong tetrahedral network of silicate glass maintains its hardness up to a glass transition temperature where the polymer is completely degraded, which implies formation of an interdiffusion layer between glass and polymer at room temperature is almost impossible. Therefore, the most typical way of interface processing would be to utilize intermolecular interactions between the polymer and glass elements on the surface [15]. However, it is notable that there can be various kinds of interactions for polymer–glass interfaces following types of intermolecular force encompassing from weak ‘physical’ interactions to strong ‘chemical’ bonding [16].

Atomistic modeling techniques have proved to be powerful tools for studying the mechanisms of interfacial behavior in molecular scale [17,18,19,20,21,22,23]. Density functional theory (DFT) provides electronic structure of molecules, which gives us an intrinsic information for chemical affinity at the interface. In addition, molecular dynamics (MD) simulation describes a model on the length scale from nanometer to micrometer, and thus both conformational changes of polymers and adhesion/detach process of a film can be analyzed. Chemical bonding and physical interactions mentioned above are often referred to as ‘bonded’ and ‘non-bonded’ interactions in the sense that physical interactions do not ‘tie’ two atoms specifically, rather gather all atoms of the group loosely [16,24]. Non-bonded interactions usually include van der Waals forces, polar interactions including hydrogen bonds, and Coulomb interaction. Often, interfacial adhesion and relevant failure behavior are not easy to understand because the above interactions contribute to the adhesion together. MD simulation can decompose energy terms of non-bonded interactions to track their separate roles for adhesion strength. In this way, it is able to consider possible factors which affect the adhesion such as effects of rigidity of the polymer film and ratio of polar functional groups. Effects of adhesion measurement methods can be also analyzed through simulated adhesion tests with various modes [25,26,27,28]. Meanwhile, the bonded interaction includes strong covalent and ionic bonding. For example, one can form covalent bonding between silane end groups of the specially prepared polymer and silanol groups on the glass surface to greatly improve interfacial adhesion. DFT calculations for such interfaces can reproduce precise steps of bonding formation under hydrolysis and condensation process, and the resultant bonding strength can be also calculated [29]. MD simulation clarifies nanoscale characteristics of the interface, such as the adhesion strength, thickness, and surface density of the polymer film [30]. Furthermore, for both cases of bonded and non-bonded interaction, atomistic modeling can artificially control the extrinsic conditions, such as the surface morphology of the glass substrate and relative humidity, to study their unique effects on the adhesion [29,30,31,32,33].

In this review, recent atomistic modeling works with a focus on the interfacial interaction between polymer coating films and silicate glass are presented. In Section 2, core computational details for building of interfacial systems by MD simulation techniques are described, and useful modeling techniques to reproduce adhesion test are explained. With two categories of non-bonded and bonded interaction, the interfacial mechanism is reviewed in Section 3. In the case of non-bonded interactions, underlying mechanisms of adhesion and failure behavior are discussed for various types of polymers ranging from homopolymer, copolymer, natural polymer, and surfactant. Interfacial properties are investigated by controlling relevant factors, such as the rigidity of the film, polarity of the functional groups, and adhesion test modes. In the case of bonded interactions, the mechanism of covalent bonding formation between the polymer and the glass surface is summarized, and film properties of the polymer coating are estimated by analyzing adhesion strength at the interface. In Section 4, the effects of two extrinsic conditions of surface morphology and relative humidity on the interfacial properties are reviewed in detail.

## 2. Computational Methodology

The basic procedures of MD simulation applied to the works in this review are as follows: bulk silica structures are generated with melt and quench method, and then the surface is created by considering hydroxylation density and surface roughness. Initial structures of polymers are prepared and relaxed with thermal annealing and combined with silica surface to form interfacial system. Interfacial properties are analyzed after the interfaces are equilibrated, and adhesion between two surfaces are measured [27,28,30,31,33,34]. In this section, a main force field used in the MD works is introduced, and simulated pulling, peeling, and sliding tests are described with Steered Molecular Dynamics (SMD). Techniques for building of ‘rough’ glass surface and dynamic bond creating/breaking method are briefly explained.

### 2.1. Force Field

Interface force field employs the same functional form of common harmonic force field as PCFF, COMPASS, CHARMM, AMBER, GROMACS, and OPLS-AA, and it has been extended to organic–inorganic and inorganic–biomolecular interfaces [24]. Parameters are developed by understanding of physical and chemical properties and validated with experimental and ab initio results for surface phenomenon. Especially, it successfully predicts adsorption properties of glass–polymer interface [35], and hence adopted in this work. Among the several forms of interface force field, PCFF interface force field, which is an extension of the well-established PCFF force field is adopted, and its functional form is in below.

(1) $$\begin{array}{cc} E_{pot}= & \sum_{ij,\;nonbonded\;1,2\;and\;1,3\;excluded}\varepsilon_{ij}\left[2{\left(\frac{\sigma_{ij}}{r_{ij}}\right)}^{9}-3{\left(\frac{\sigma_{ij}}{r_{ij}}\right)}^{6}\right]\\ & \;+\frac{1}{4\pi \varepsilon_{0}}\sum_{ij,\;nonbonded\;1,2\;and\;1,3\;excluded}\frac{q_{i}q_{j}}{r_{ij}}\\ & \;+\sum_{ij,\;bonded}k_{r,ij}{\left(r_{ij}-r_{0,ij}\right)}^{2}+\sum_{ijk,bonded}k_{\theta ,ijk}{\left(\theta_{ijk}-\theta_{0,ijk}\right)}^{2} \end{array}$$

More details on the formulation and parameterization of individual terms can be found in the original paper by Heinz et al. [24].

### 2.2. Pulling, Sliding, and Peeling with Steered Molecular Dynamics

Adhesion behavior of polymer on glass depends on the chain rigidity, binding nature, and detachment process. In this regard, three methods of pulling, peeling, and sliding tests are implemented with steered molecular dynamics (SMD) simulations and applied to the PI–glass [25,27,31] and SPFPE–glass interface systems [30].

SMD can calculate the potential of mean force (PMF) by employing Jarzynski’s equality, which relates the equilibrium quantity (PMF) to the non-equilibrium process [36,37]. In the SMD method, a virtual spring is considered to connect atoms in the structure and dummy atoms, which are displaced with a constant velocity from the atoms. Thus, force is applied on the atoms to detach them from each other, and the generated force due to the constant velocity is defined as follows:

(2) $${\vec{F}}_{spring}=-\nabla U_{spring}$$

where *U_spring_* is the generated potential with a harmonic spring type model, and it is defined as follows:

(3) $$U_{spring}=\frac{1}{2}k{\left[vt-\left(\vec{R}\left(t\right)-{\vec{R}}_{0}\right)\cdot \vec{n}\right]}^{2},$$

where *k* is the spring constant of a virtual spring, *v* is the constant pulling velocity, *t* is the time, $\vec{R}\left(t\right)$ and ${\vec{R}}_{0}$ are the current and initial positions of the center of mass of the pulled atoms, and $\vec{n}$ is the pulling direction. Therefore, work performed (*W*) during the pulling process is calculated as follows:

(4) $$W=\int_{r=R_{0}}^{r=R_{f}}\nabla U_{spring}\cdot d\vec{r}$$

where *R*_0_ and *R_f_* are the initial and final positions of the center of mass. By using Jarzynski’s equality, PMF is computed as follows:

(5) $$\mathrm{PMF}=-\frac{1}{\beta }log\langle e^{-\beta W}\rangle {}_{ensemble}$$

where $\beta =\frac{1}{k_{B}T}$ with the Boltzmann constant *k_B_* and system temperature, T, and angular brackets indicating an ensemble average of the given values. During the SMD simulations, temperature and spring constant are 300 K and 100 kcal/(mol·Å^2^), respectively. Constant velocity is then applied after testing several velocities in the range 1–100 m/s to obtain the converged behavior of the PMF.

**Figure 1:**
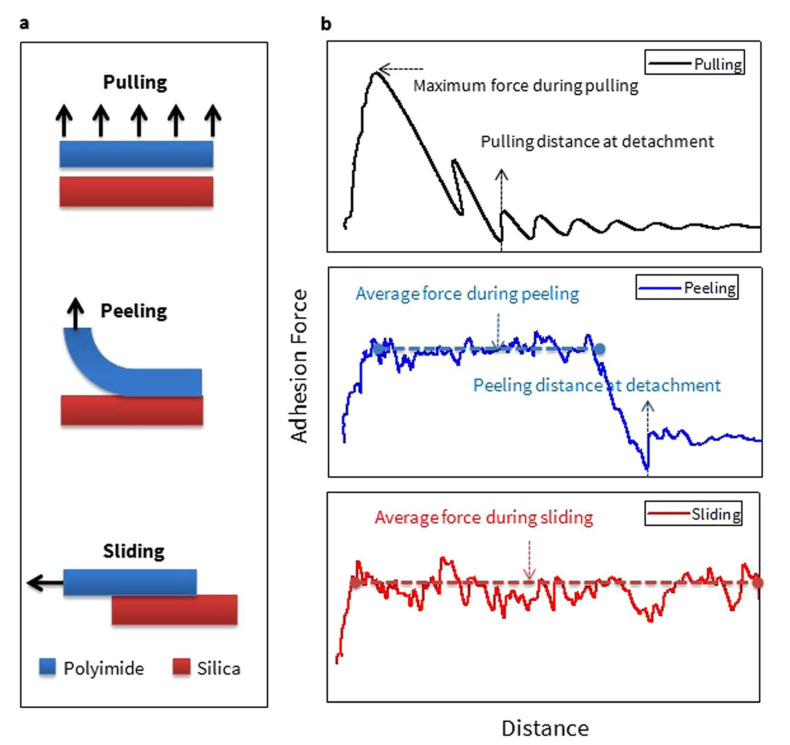
(**a**) The schematic view and (**b**) representative properties for characterizing the adhesion properties from each of adhesion testing modes (adapted with permission from Reference [27], Copyright 2017 Springer Nature).

Figure 1 shows schematics of the three methods, along with representative properties during the simulation test. Depending on deformation direction and area, the adhesion mechanism is determined. In the case of a pulling test, uniform velocity is applied to theopposite direction for the polymer and glass, and maximum force and pulling distance for the detachment can be characterized for various interfaces. Peeling test is basically the same as pulling test except the velocity is only applied to a smaller region, and a plateau region in the peeling process is interpreted as homogeneous adhesion between two layers. Adhesion energy is defined as the maximum PMF value divided by the projected interfacial area, and its convergence behavior is used to find an optimal pulling velocity. When the velocity is faster than the converged value, the average PMF is usually overestimated, which implies that the process is no longer in equilibrium. However, adhesion energy from peeling process largely depends on the peeling width, and thus it is hard to determine for the peeling process. The longer peeling width leads to shorter plateau area, which results in smaller PMF for detachment. For the sliding test, a uniform velocity is applied to the polymer atoms with shear stress on both sides. In this case, due to the periodic boundary condition on the sliding direction, the interface of two layers remain in contact with each other, and hence a detachment distance cannot be determined. Instead, the magnitude of average force during sliding can provide insight regarding the adhesion for various interfaces.

### 2.3. Generation of Surface Roughness

**Figure 2:**
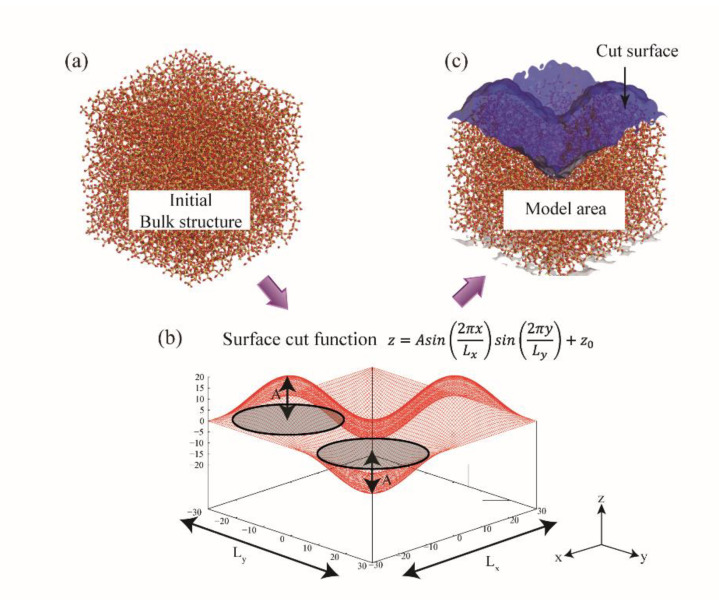
Schematic procedure of generating rough surface. (**a**) Initial bulk glassy silica. (**b**) Predefined surface with sine function, $z=A\;\sin\frac{2\pi x}{L_{x}}\sin\frac{2\pi y}{L_{y}}+z_{0}$. (**c**) Prepared rough glassy silica surface; blue shaded area represents cut surface by the sine (adapted with permission from Reference [31], Copyright 2017 American Chemical Society).

Experimentally, the arithmetic average of height variation of the surface compared to a reference plane, *R_a_*, is usually measured and used as a roughness parameter. However, it does not contain any information regarding the roughness shape, and hence a deterministic surface roughness including both amplitude and spacing parameters is a good choice for the systematic analysis of roughness shape dependence. As shown in Figure 2, predefined parameters can be used to create surface roughness by cutting a bulk material [31]. Firstly, a surface shape with a specific mathematical function is defined, and then the initial bulk structure is cut according to the surface roughness function. The surface is created by keeping all atoms under that function. Roughness can be adjusted by modulating the predefined surface function, and, as one example, a sinusoidal function is presented, as follows, by varying amplitudes and spacing [38]:

(6) $$z=A\;\sin\left(\frac{2\pi x}{L_{x}}\right)\sin\left(\frac{2\pi y}{L_{y}}\right)+z_{0}$$

where *A*, *L_x_*, *L_y_*, and *z*_0_ represent roughness amplitude, spacing along each axis, and the average chosen height, respectively. Various amplitudes and spacings can be considered to analyze the effect of each parameter. To mimic bulk material response during the simulation, atoms at the bottom of the surface should be fixed during simulation run.

### 2.4. Bond Creating/Breaking

**Figure 3:**
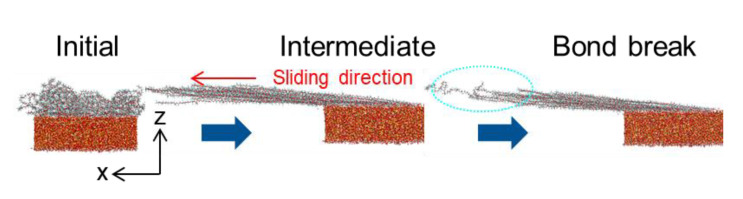
Snapshot of dynamic bond breaking during sliding process for SPFPE–silica interface (adapted with permission from Reference [30], Copyright 2019 American Chemical Society).

In order to accurately predict interfacial properties in the atomistic simulation, it is required to model dynamic breaking of bonds within the materials. Especially, failure modes for bonded interaction can only be predicted with bond breaking. Reactive force field or ab initio molecular dynamics (AIMD) can treat bond formation and breaking for molecules, but it requires huge computational resources, which greatly limits the system size and simulation time. In this regard, a combination method of density functional theory (DFT) and classical molecular dynamics is developed [30]. Bond dissociation energies (BDE) for any bond considering its neighboring environment can be obtained from DFT. Molecular dynamics simulation is then conducted with the INTERFACE force field which accurately predicts interfacial properties between polymer and silica [24]. In the INTERFACE force field, the bonding energy contribution is represented by using the quartic harmonic approximation with the following form:

(7) $$E=K2\times {\left(R-R_{eq}\right)}^{2}+K3\times {\left(R-R_{eq}\right)}^{3}+K4\times {\left(R-R_{eq}\right)}^{4}$$

where *K*2, *K*3, and *K*4 are bond constants, and *R_eq_* is the equilibrium bond distance. Using this equation, we find a value of *R* when *E* equals the *BDE* computed by DFT, where the bond is explicitly broken in the simulation. This method is applied to predict SPFPE detachment process [30], and Figure 3 shows dynamic bond breaking during sliding process. Adopting this method, relatively larger systems can be modeled with accurate bond dissociation information.

## 3. Mechanism Analysis on Interfacial Interactions

### 3.1. Non-Bonded Interaction

In the interfacial system based on non-bonded interaction, it is obvious that different kinds of non-bonded interactions critically determine the adhesion strength. For example, polar interactions between permanent dipoles, such as hydroxyl groups and carboxylic acid groups, are relatively strong, and hydrogen bonding between protons and highly electronegative anions is the strongest type of interaction [11,16]. Including the polar groups, chemical composition of a molecule at the level of monomer unit affects interfacial interaction. In the case of polymerized chains, flexibility of the individual chain may be an important factor because it comprises a chain-to-chain interaction of the film and determines the degree of chain conformation, which finally changes adhesion and failure behavior. Meanwhile, even for the same interfacial system, different adhesion measurement methods result in different adhesion values and relevant failure mechanism. In this subsection, several atomistic modeling works on the polymer-glass interface based on non-bonded interaction are reviewed with the relevant factors, such as flexibility of polymer material, polarity of the functional groups for both polymer and glass parts, and modes of adhesion test.

**Figure 4:**
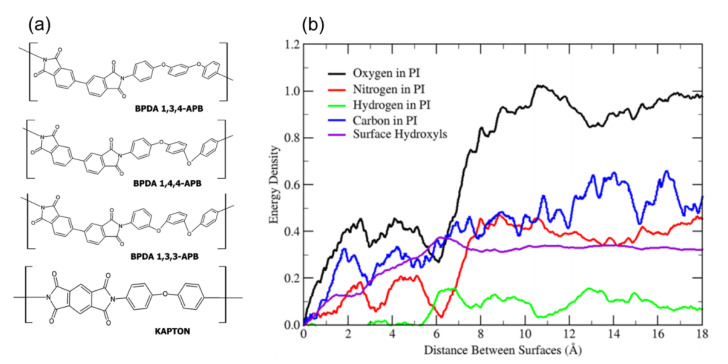
(**a**) Chemical structure of polyimide monomers for three BPDAs and Kapton. (**b**) Energy density profile for atomic subgroups in the polyimide (adapted with permission from Reference [25], Copyright 2016 American Chemical Society).

Recently, adhesion behavior of polyimide (PI), which is a rigid type of homopolymer, on the glass surface was intensively investigated from a single monomer to a polymer film [25,26,27,31,34]. Goyal et al. studied fundamental adhesion behavior of PI monomer on the glassy silica surface [25]. Effects of PI chemistry, surface hydroxylation density and crystallinity of the glass on the adhesion strength were considered as a main focus of the report. Four kinds of monomers were prepared on the silica glass, as shown in Figure 4a. Three of them share 3,3′4,4′-biphenyl tetracarboxylic dianhydride (BPDA) with one of three different APB isomers: 1,3-bis(3-aminophenoxy)benzene, 1,3-bis(4-aminophenoxy)benzene, or 1,4-bis(4-aminophenoxy)benzene, and the other one is pyromellitic dianhydride 4,4′-oxidiphenylamine (Kapton). The pulling test was applied to detach the monomer from the glass surface. In the regard to the effect of PI chemistry, adhesion strength for BPDAs with three different APB linkages were similar with each other, whereas Kapton showed 20% higher adhesion than BPDAs. As shown in Figure 4b, the major contributor for the adhesion energy is oxygen in the PI monomers, and thus one concludes that the molecular origin of the difference in adhesion is a higher oxygen density of Kapton than that of BPDA. When the adhesion of fully hydroxylated and non-hydroxylated silica surface is compared, monomer with hydroxylated glass surface showed 20% higher adhesion than non-hydroxylated one. It was understood that hydrogen bonds between oxygen or nitrogen in the PI monomers and hydroxyl groups on the glass surface resulted in higher adhesion energy. Moreover, adhesion for amorphous silica case was more than 30% higher than that for crystalline silica, which can be also explained with hydrogen bonding term. Due to randomness of the surface sites for amorphous silica, chances to form hydrogen bonding between PI monomer and glass were increased.

**Figure 5:**
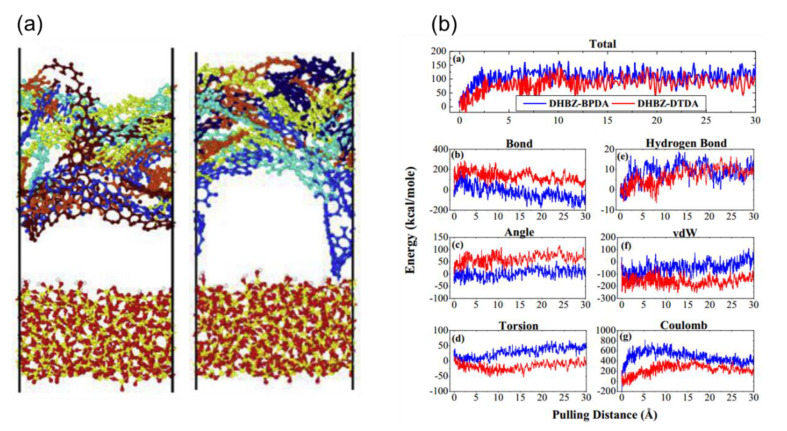
(**a**) Snapshots for deformation of polyimide chain: DHBZ–BPDA (left) and DHBZ–DPDA (right). (**b**) Decomposition of energy terms and their evolution as a function of pulling distance (reprinted from Reference [26], Copyright 2016, with permission from Elsevier).

In addition to the monomer behavior of PI, adhesion of PI film on the glass was studied by Min et al. with a focus on flexibility of the film [26]. One can expect that flexibility of the chain may give different degrees of conformation during adhesion test, which finally affect the adhesion together with van der Waals interaction at the interface. As indicated in Figure 5a, authors found that more flexible PI (3,3′-dihydroxy benzidine with 4-(2,5-dioxotetrahydrofuran-3-yl)-1,2,3,4-tetranaphthalene-1,2-dicarboxylic anhydride, i.e., DHBZ–DTDA) shows larger chain deformation than rigid PI (3,3′-dihydroxy benzidine with 3,3′,4,4′-biphenyl tetracarboxylic dianhydride, i.e., DHBZ–BPDA) during pulling test which resulted in smaller pulling force yet longer detachment distance. Further analysis was performed for the interface by decomposition of energy terms as shown in Figure 5b.

The result shows that, for both DTDA and BPDA, intramolecular energy from C–C bonds in the chain initially increases at the early stage of pulling, but readily reduces to zero and even becomes negative. It implies that bond energy initially plays a role of resistance against the pulling force but stops contributing to adhesion as soon as the film is relaxed after detachment. On the other hand, it turns out that non-bonded Coulomb interaction critically contributes to the adhesion energy throughout the whole process of pulling. This is especially noticeable in the case of rigid PI (BPDA) because strong pulling force is required for detachment the whole film at the same time due to its rigidity.

**Figure 6:**
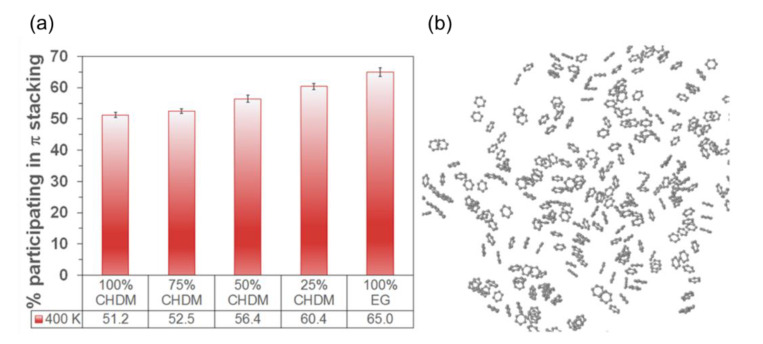
(**a**) Ratio change of aromatic rings participating in π stacking; (**b**) Rings forming π stacking for 100% CHDM case (adapted with permission from Reference [35], Copyright 2016 American Chemical Society).

In addition to a homopolymer, adhesion of copolymer on the glass surface was studied by Hanson et al. [39]. In this work, adhesion of polyester copolymer on soda-lime glass was calculated depending on the composition ratio of ethylene glycol (EG) to cyclohexanedimethanol (CHDM). They report that the adhesion of copolyester film increases as the ratio of EG increases; however, there were no changes in the number of polar functional groups in the copolymer and surface hydroxylation of the glass. Figure 6 shows that the amount of aromatic π stacking between the chains increases as the EG ratio increases. It is thought that this higher rigidity due to denser π stacking leads to an increase of adhesion, which is another example in which the flexibility/rigidity of the polymer itself affects the interfacial adhesion.

**Figure 7:**
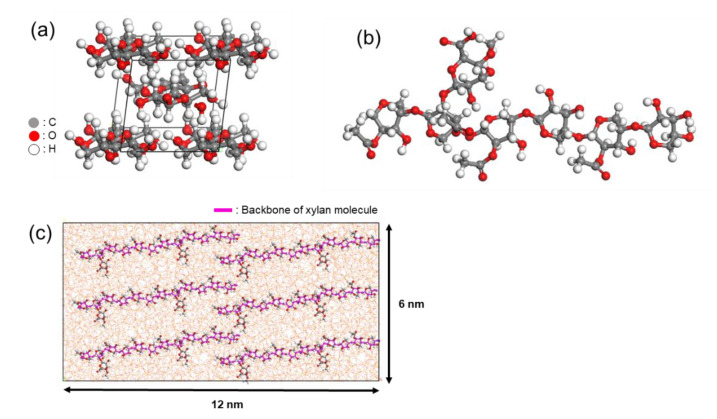
(**a**) Monoclinic crystalline structure of cellulose-Iβ. (**b**) Structure of Glucuronoxylan (xylan) hemicellulose molecule. (**c**) Bottom view of stretched configuration of 6 xylan molecules under the surface of cellulose film.

Park et al. investigated the interface between glass and paper materials, wherein the paper belongs to the family of natural polymers and its chemical structure is a combination of rigid and flexible polymeric parts [32]. Figure 7a,b describes chemical structures of two polymeric ingredients of the paper material. The main composition consists of rigid cellulose microfibrils which have a crystalline structure, while around 10 wt% of the composition is xylan hemicellulose (abbreviated as xylan), which is a flexible molecule with shorter chain length [40,41,42]. As shown in Figure 7c, the whole structure of the paper film can be described in such a way that xylan molecules are attached on the surface of the cellulose microfibrils with stretched configuration [43]. These two ingredients commonly possess polar functional groups such as hydroxyls or carboxylic acids, and thus one can expect that polar groups as well as flexibility of the paper material affect the adhesion between paper and the silica surface. Compared to cellulose-only case, adhesion calculations show that inclusion of only a few xylan molecules on the cellulose film leads to remarkable enhancement of the adhesion force. This result has two implications: (i) the average strength of hydrogen bonds induced by hydroxyl groups in the cellulose film is not strong, and (ii) only a few carboxylic acid groups in the xylan molecules make strong hydrogen bonds with hydroxyl groups on the silica glass. Furthermore, the high flexibility of xylan molecule enables large deformation which leads to high adhesion force for the cellulose–xylan composite case.

**Figure 8:**
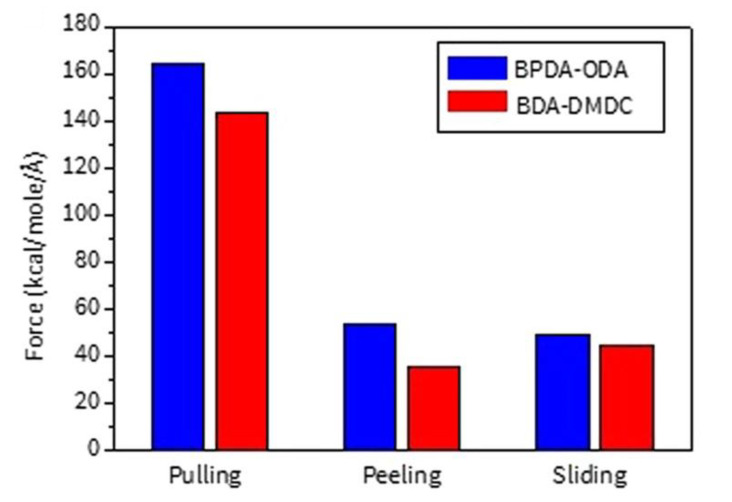
Comparison of adhesion for polyimides between pulling, peeling, and sliding modes of adhesion test (adapted with permission from Reference [27], Copyright 2017 Springer Nature).

**Figure 9:**
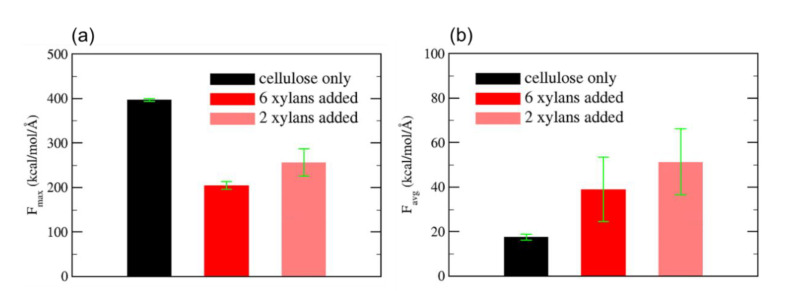
Comparison of adhesion force between cellulose and xylan-cellulose composite cases during (**a**) pulling test and (**b**) sliding test.

One can expect that precise value of adhesion may be different depending on the kind of adhesion measurement methods. Min et al. prepared two kinds of interfacial systems between aromatic and aliphatic kinds of PI films and the silica glass [27]. Aromatic PI, biphenyltetracarboxylic dianhydride (BPDA), and 4,4′-oxydianiline (ODA) are denoted as BPDA–ODA, and aliphatic PI, 1,2,3,4-butanetetracarboxylic dianhydride (BDA), and 4,4′-diaminodicyclohexylmethane (DMDC) are denoted as BDA–DMDC. Authors applied three kinds of adhesion test modes such as pulling, peeling, and sliding to compare adhesion strengths and the features of dominant interfacial interactions for each mode between them. As shown in Figure 8, for both BPDA–ODA and BDA–DMDC, they found that adhesion force for the pulling mode is three times higher than that for peeling and sliding modes, while it was comparable between peeling and sliding modes. It can be pointed out that the effective volumes where chains experience deformation are different from each other; namely, the volume in the pulling mode is so large that all of the chains in the whole PI film region undergo conformational change whereas volumes for the peeling and sliding modes are only the front and interfacial region of the film, respectively. Authors also studied effect of PI structure on the adhesion force depending on adhesion modes. Due to the presence of charge transfer complex, BPDA–ODA basically shows higher rigidity than BDA–DMDC and thus, adhesion force of the former is higher than the latter. It is noticeable that peeling mode shows larger difference in the adhesion force than other two modes. One can understand that only peeling mode makes the film to be highly bent close to right angle which leads to significantly high pulling force for rigid BPDA–ODA. On the other hand, pulling and sliding modes do not induce such a large conformational change, and thus the difference in adhesion between two PI films is relatively small. One can understand that only peeling mode makes the film to be highly bent close to right angle which leads to significantly high pulling force for rigid BPDA–ODA. On the other hand, pulling and sliding modes do not induce such a large conformational change, and thus difference in adhesion between two PI films is relatively low. Park et al. considered relatively flexible random copolymer films which possess both polar and non-polar functional groups to calculate adhesion on the silica glass by means of pulling and sliding test modes [33]. The result shows that adhesion force for pulling test mode is 40% higher than that for the sliding mode. It is thought that the difference in adhesion is not that significant compared to the above two cases because of two points: (i) Current copolymer films are so flexible that they show a cohesive failure during pulling test, and thus they are more easily and severely deformed against the shear compared to the above cases. (ii) In the copolymer case, some polar functional groups exposed to the interface strongly interact with hydroxyl groups on the silica surface, which finally contribute to the adhesion at some extent. It is also worth revisiting the paper–silica interface case and compare the above results with it [32]. Figure 9 shows an overall comparison of adhesion forces of cellulose and cellulose–xylan composite on silica glass using pulling and sliding tests. Especially, the black bars represent cellulose–silica interface, and adhesion force for the pulling test was about 20 times higher than that for the sliding test. This big difference in adhesion can be attributed to extremely high rigidity of glucose chain in the cellulose film while an abundant amount of surface hydroxyl groups is only a minor factor. However, a comparison of 6 and 2 xylans-added cases between pulling and sliding tests shows that the difference in adhesion is reduced approximately from 200 to 40 kcal/mol/Å and from 250 to 50 kcal/mol/ Å, respectively. It implies that the difference in adhesion is significantly reduced from 20 times to only 5 times. This is because deformations of xylans effectively dissipate free energy to lower a maximal pulling force during pulling, whereas we cannot expect such a large deformation of xylans in the sliding test.

### 3.2. Bonded Interaction

Since silica surfaces are covered with hydroxyls, siloxane covalent bonds can be formed between hydroxyl groups on the glass surface and functional end groups of the polymers. Created bonds anchor the polymer on glass surface tightly to form an interface based on bonded interaction, and interfacial properties are different from the case of non-bonded interactions. Not only the functional end groups, but also the remaining part of molecules affect properties of the polymer film such as thickness, molecular density, and adhesion on the silica surface. Experimentally, it has been found that topological characteristics and mechanical properties of alkyltrichlorosilane (ATS) on the Si(100) surface strongly depend on the atomic structure of the silane groups due to the steric hindrance between alkyl chains [44], and that the morphology of gold nanoparticles (AuNPs) can be controlled by bonding structures between silane groups of aminopropyltriethoxysilane (APTES) and glass surfaces [45]. Surface reactivity, molecular density, and adhesion property of perfluoropolyether (PFPE) have been recently studied, using atomistic modeling, and are introduced in this review.

#### 3.2.1. Silane Functionalized Perfluoropolyether (SPFPE) and Siloxane Bond Formation

**Figure 10:**
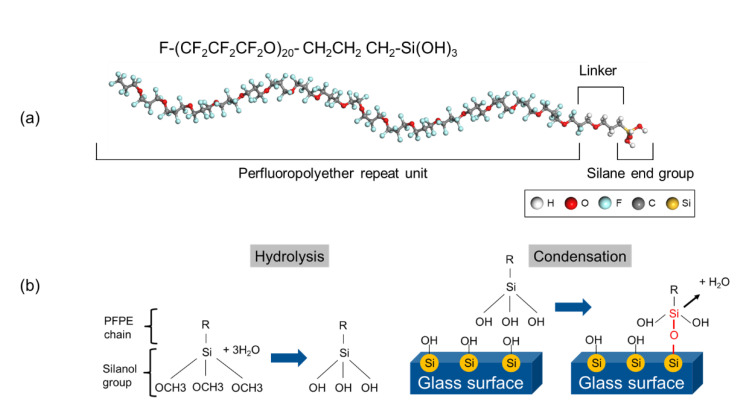
(**a**) Molecular structure of silane functionalized perfluoropolyether (SPFPE) single chain. (**b**) Schematics of surface reactions between silica surface and SPFPE (adapted with permission from Reference [30], Copyright 2019 American Chemical Society).

A molecular structure of SPFPE consists of perfluoroether chain and functional end group as shown in Figure 10a. Anti-fouling properties come from perfluoroether chains, and functional end groups anchor the molecules to the glass substrate. As a functional end group of PFPE-derived molecules, alkoxysilanes are frequently adopted because the hydroxyl groups on the glass surface interact with the alkoxy groups of the silanes through hydrolysis and condensation reactions, as shown in Figure 10b [46,47,48]. Hydrocarbon- or fluorocarbon-silane molecules generally form self-assembled monolayers (SAM) on silica surfaces due to the hydrophobicity of both chains, which induce close packing of polymers on hydrophilic silica surfaces [49,50,51,52]. On the other hand, oxygen atoms in the perfluoroether repeat units enhance the affinity of PFPE chains on the hydroxylated silica surface, and thus the driving force for the packing of SPFPE is reduced, and lower molecular density can be expected [29].

The procedure of siloxane bond formation during SPFPE deposition on silica surface is shown in Figure 10b, which is a general surface reaction for silane functionalized molecules. When SPFPE molecules approach to the surface, silanol branches of the silane group can interact with a hydroxyl groups on the silica surface. As a result, siloxane bonds are formed through condensation reaction, and if there are multiple hydroxyl groups on the silica surface, remaining silanol branches of SPFPE molecules can form additional siloxane bonds. Although a single SPFPE molecule contains three silanol branches, it is revealed that only a single siloxane bond can be formed on the silica surface from activation and reaction energy calculations using density functional theory [29]. This is because geometrical deformation of the silane tetrahedron acts as an energy barrier for the additional siloxane bond formation, and pre-existing hydrogen bonds on the silica surface further enhance the energy barrier for the bond formation.

#### 3.2.2. Film Property and Adhesion of SPFPE

**Figure 11:**
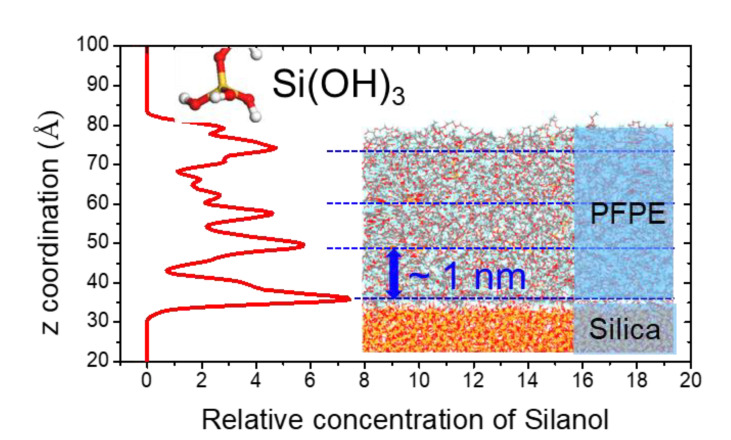
Concentration profile of silanol of SPFPE along with *z*-axis on silica surface (adapted with permission from Reference [30], Copyright 2019 American Chemical Society).

Among the various deposition techniques such as spray coating, dip-coating, and physical vapor deposition methods, thermal evaporation deposition was reproduced with sequential SPFPE insertion and relaxation process by Lee et al., and film properties and interfacial behavior of SPFPE are analysed [29,30]. It is known that surface coverage for self-assembled monolayers (SAM) ranges from 2.5 to 5.3 molecules/nm^2^ [49,53,54,55], and molecules are aligned with a perpendicular orientation on silica surface. On the other hand, the orientation of adsorbed SPFPE on silica surface is parallel to the surface plane, and the estimated surface coverage of SPFPE on the silica surface is only 0.31 molecules/nm^2^, which means a single SPFPE molecule covers a much larger surface area than the conventional hydrocarbon- or fluorocarbon-silanes [29]. It is also observed that formation of crosslinked structures between the adjacent SPFPE molecules is inhibited, and parallel orientation not only reduces surface coverage but also induces thin thickness of the SPFPE layer on the silica surface. As a result, deposited SPFPE layer on silica surface exhibits 1 nm thickness where the bottom layer is bound to the silica surface as shown in Figure 11 [29,30,56]. Molecular permeability of SPFPE is analysed by means of mean square displacement (MSD), and calculated results revealed that average displacement of the SPFPE is less than 2.5 Å in z direction, which implies additional SPFPE molecules hardly reach the silica surface. Therefore, the additional molecules cannot be bound to the silica surface, and hence remain as several stacks of SPFPE layers, as shown in Figure 11. Thickness of SPFPE is observed as 1 nm on rough surface as well, and hence it is expected that nanoscale roughness of the glass substrate can highly affect the interfacial properties.

The durability of SPFPE on the silica surface is primarily influenced by the reaction between the silane of SPFPE and the hydroxyl groups on the surface. The sliding mode of adhesion is applied to investigate adhesion strength of SPFPE, and siloxane bonds are determined as a major source of adhesion strength [30]. Therefore, the bound layer to the surface is key for the durability of SPFPE, while the additional SPFPE layers on top of the bound layer can be easily detached from the surface during the sliding process. The predicted molecular density of SPFPE on the silica surface is 15 times lower than the conventional hydroxylation density of silica, 4.6 OH/nm^2^. Therefore, unlike polyimide, the impact of surface hydroxylation density of silica is limited for SPFPE adhesion [25,31]. Instead, modulation of the SPFPE molecular structure can be an effective way to enhance adhesion, and reducing molecular weight, as one example, increases the adhesion strength by enhancing SPFPE molecular density. However, it is noteworthy that a reduced PFPE chain can also decrease the antifouling performance, and thus a comprehensive study is required to find optimal weight for both higher adhesion and antifouling performance.

## 4. Extrinsic Conditions Affecting Interactions

### 4.1. Surface Morphology: Effect of Nanoscale Roughness on the Adhesion

Nanoscale roughness exhibits critical role for the adhesion of interfaces with both non-bonded and bonded interactions. For the non-bonded interaction, hydroxylation density on silica surface was one of the key parameters for adhesion of PI [25], and thus it is a practical way to enhance adhesion by increasing hydroxyl density with surface roughness. For the bonded interaction, surface reaction at the interface determines molecular density and hence, roughness is a parameter of interest that impacts adhesion. As described in Section 2.3, ordered roughness is defined by amplitude and spacing (Figure 2) [38], and the effects of individual parameters on the adhesion are investigated for both PI and SPFPE adsorption on the silica surface [30,31].

**Figure 12:**
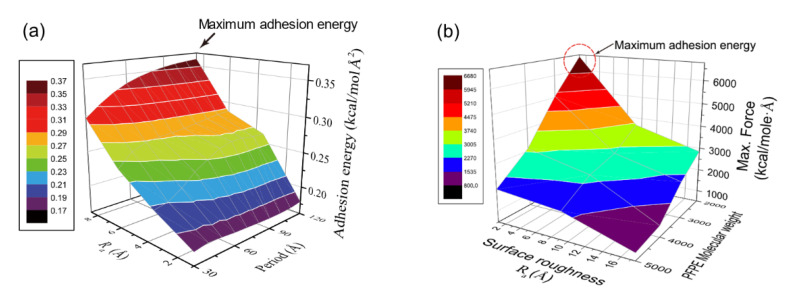
Variation of adhesion energy for (**a**) PI–glass and (**b**) SPFPE–glass interface (adapted with permission from References [30,31], Copyright 2019 and 2017 American Chemical Society).

Figure 12a shows adhesion energy variation for PI–glass interface depending on surface roughness, and roughness amplitude (R_a_) exhibits the highest impact on the adhesion. Roughness spacing corresponds to the period of ordered roughness, and it is important to mention that the impact of amplitude is also determined by the roughness period [31]. As the roughness period decreases, un-contacted area between PI and glass increases with formation of vacant pores, and hence effect of amplitude on the adhesion is reduced. As roughness period increases, the two surfaces are well-attached to each other, and adhesion energy is maximized when the period is the longest (see Figure 12a). However, a further increase of spacing implies reduction of the surface area, and thus there exists an optimal roughness spacing for maximal adhesion strength. In addition, the energetic contribution to the adhesion is analyzed by energy decomposition during the pulling process, and it is revealed that not only is there an increase of hydrogen bond energy but also the increase of Coulomb energy contributes to the adhesion energy variation by roughness. The contribution of Coulomb energy becomes more significant when roughness is higher [31].

For the bonded interaction of SPFPE, mode of sliding process is applied, and force for bond breaking is measured varying surface roughness and molecular weight of SPFPE [30]. As shown in Figure 12b, effect of roughness amplitude is different from that for PI. As the roughness amplitude increases, the maximum force decreases which implies reduction of adhesion strength. Due to the low entanglement and 1 nm thickness of SPFPE, only the bound SPFPEs have an impact on the adhesion. Therefore, when there is roughness on the surface, not all SPFPEs are involved in the sliding process, and hence the adhesion strength decreases with increasing roughness. Adhesion energy trend with roughness spacing is similar to that for the non-bonded interaction. With a short roughness spacing, there exist vacant pores at the interface and molecular density reduces. However, when the roughness spacing is long enough to adsorb molecules without pores, adhesion increases due to the increased surface area. For the SPFPE adhesion, molecular density of bound SPFPE is a primary parameter, and the reduction of molecular weight can be a good way to increase adhesion strength, as shown in Figure 12b.

### 4.2. Humidity

Humidity is one of the dominant environmental factors which hugely affects interfacial properties because of its ability to modify the surface structure of glass, as well as the interface between coating material and glass. It is known that only a few water molecules can induce hydroxylation of the glass surface, and water amounts corresponding to 15% relative humidity are enough to form a hydrated surface with a monolayer of water [57]. Furthermore, water molecules can diffuse into the polymer film and modify flexibility of the chain, thereby affecting adhesion too; therefore, the effect of humidity on the interfacial properties is of great importance.

#### 4.2.1. Comparison of Dry and Wet Condition

**Figure 13:**
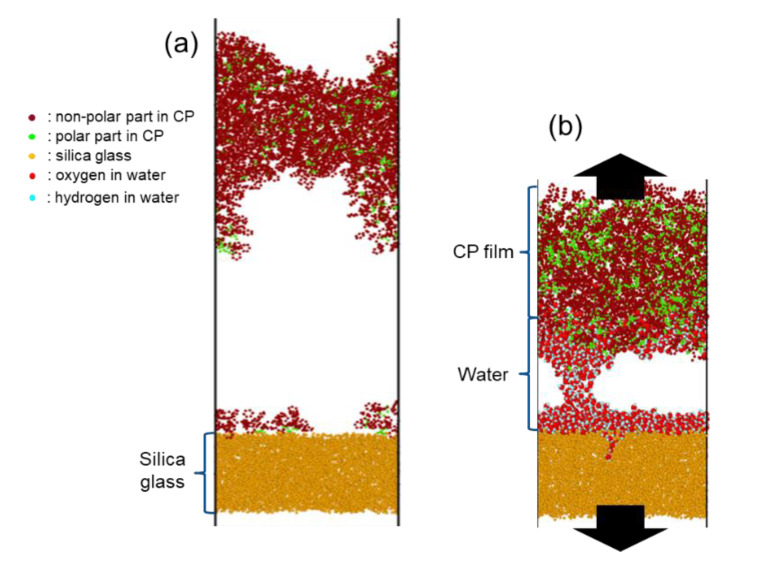
(**a**) Cohesive failure of copolymer (CP)–silica interface in the absence of water. (**b**) Adhesive failure of the interface in the presence of water during pulling test.

In the pulling simulation performed by Park et al., a shift of the failure mode is observed at the random copolymer-silica interface depending on the humidity condition [33]. Figure 13a shows that the copolymer (abbreviated as CP in the rest of the paper) film undergoes cohesive failure in the perfectly dry condition, whereas Figure 13b shows an adhesive failure in the presence of interfacial monolayer of water. Cohesive failure of the CP primarily means that its chain-to-chain interaction is weaker than the interfacial interaction with silica surface, and thus one can conclude that water molecules which were diffused inside the chain enhanced chain-to-chain interaction to overcome interfacial interaction by making the film more rigid. Adhesion energy is lowered during the shift from cohesive to adhesive failure because water molecules effectively remove the additional energy contribution for chain deformation.

**Figure 14:**
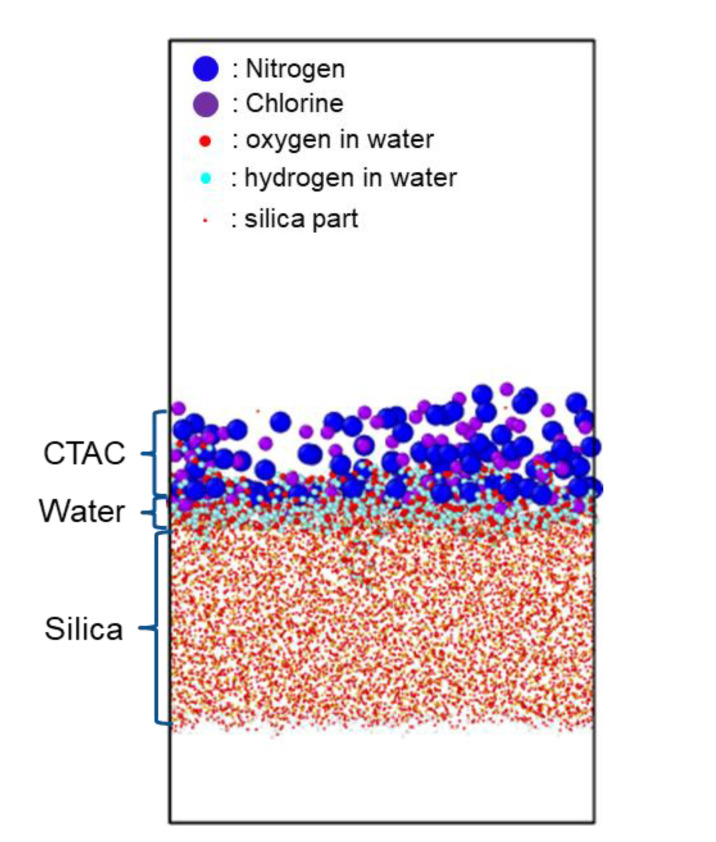
CTAC–silica interface in the presence of interfacial water layer. Tail groups of CTAC were omitted for clear visualization of the interface.

Another important class of interfaces regarding water is the surfactant–glass interface [33]. Surfactant materials-cationic surfactant, in particular—are known to form self-assembled (micellar) structures in an aqueous environment, since they consist of a hydrophilic head group with a positive charge at one end and a hydrophobic tail group which is a linear hydrocarbon chain at the other end [58,59,60,61]. In the work of Park et al., 16-hexadecyltrymethylammonium chloride (C_16_TAC, abbreviated as CTAC in the rest of the paper) was used as an example of popular cationic surfactant for the study of surfactant-glass interface [62]. When CTAC molecules are deposited onto the silica substrate, most of the head groups tend to be adsorbed on the surface, and thus the adhesion strength is mainly determined by net surface coverage of the head group on the silica surface. Now, the introduction of water significantly changes the structure of CTAC–silica interface and the adhesion strength. As shown in Figure 14, introduced water molecules penetrate the interface very well, and the head groups are located further from the silica surface, which leads to a reduction of surface coverage of the head groups. Therefore, one can expect that adhesion strength of CTAC with high coverage is also reduced. In terms of interfacial interactions, the penetration of water molecules between CTAC head groups and silica surface is easy because the basic interactions in the surfactant–silica interface are van der Waals and Coulomb interactions, which are weaker than hydrogen bonding that water forms with the silica surface. This makes a clear contrast with the CP–silica interface case above. In that case, the copolymer itself has a lot of functional groups which serve as potential sites for additional hydrogen bonding with water molecules, so that, when water is introduced, the adhesion can increase.

#### 4.2.2. Control of Relative Humidity

**Figure 15:**
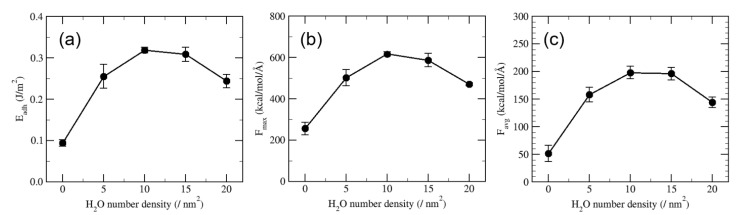
Change of adhesion level as a function of H_2_O number density: (**a**) adhesion energy, (**b**) maximum force, and (**c**) average force.

In addition to the comparison between dry and wet conditions, it is also interesting to control the amount of water molecules absorbed in the silica surface, which can be corresponding to atmospheric humidity. In the study by Park et al., the effect of gradual change of relative humidity on the adhesion of paper-silica interfaces was also studied [32]. Various amount of water molecules in the range of 0 to 80 H_2_O/nm^2^, where 5 and 20 H_2_O/nm^2^ roughly corresponds to relative humidity of 15% and 75%, respectively, were introduced to the paper-silica interface, and the adhesion mechanism was analyzed [57]. Even a low humidity of 5 H_2_O/nm^2^ which corresponds to an almost-dry condition significantly enhances adhesion between cellulose–xylan composite and silica compared to the perfectly dry condition. It is thought that this enhancement of adhesion is responsible for another set of hydrogen bonding provided by interfacial water molecules. Interestingly, as shown in Figure 15, adhesion level reached a maximum point at a humidity level of 10 H_2_O/nm^2^ and decreased beyond that humidity for both pulling and sliding tests. This implies that, as the water layer increases, the van der Waals interaction at the paper-silica interface becomes dominant, whereas the contribution of hydrogen bond to adhesion is diminished.

## 5. Conclusions

Interfacial interactions and adhesion behavior of polymer–glass interfaces were reviewed in this paper, with a focus on atomistic simulation methods. Various types of polymers, such as homopolymers, copolymers, natural polymers, and surfactants, were considered, and depending on the surface adsorption behavior, polymer-glass interactions were classified as non-bonded and bonded interactions. In the works for non-bonded interaction, three main interactions, namely van der Waals, polar, and hydrogen bonds, were reviewed, and the contributions to interfacial adhesion energy were extensively analyzed. It was revealed that the dominant interaction for adhesion is hydrogen bonding due to hydroxyl groups from both the polymer molecules and the glass surface. In addition, it was found that the flexibility of the polymer chain and modes of adhesion test can affect adhesion and failure behavior at the interface. In the case of bonded interactions, creation of covalent siloxane bonds between silane groups in the polymer and hydroxyl groups on the glass surface are critical for strong interfacial interaction. A detailed mechanism of covalent bond formation was described, and adhesion properties, along with molecular density analysis, were reviewed with an example of SPFPE. One finds that parallel orientation of SPFPE is observed and only a single siloxane bond is formed among three silanol groups in the branch of SPFPE. Therefore, molecular density and thickness of the film are very low compared to the conventional self-assembled monolayer molecules. It is suggested that one effective way to enhance adhesion is to increase the molecular density of SPFPE rather than increasing the number hydroxyl groups on the silica. Besides interfacial interactions, external conditions, such as the surface morphology of the glass substrate and relative humidity, yield significant effects on the interfacial adhesion. For example, modulation of amplitude of surface roughness is most critical to the adhesion regardless of bonding type. In addition, the introduction of water molecules at the interface not only forms additional amounts of hydrogen bonds but also makes the polymer film more rigid, and thus the level of adhesion can be drastically different compared to the interface in the absence of water. In summary, comprehensive insights into the interfacial bonding mechanism of adhesion and failure behavior obtained from computational studies can be used for surface engineering purposes. It is also possible to extend such methodologies and concepts to other kinds of polymer-glass interfacial systems, as well as to understand adhesion of organic-inorganic interface in general.

## Data Availability

All data are contained in the manuscript.
